# Effects of placebo administration on immune mechanisms and relationships with central endogenous opioid neurotransmission

**DOI:** 10.1038/s41380-021-01365-x

**Published:** 2021-10-29

**Authors:** Alan Prossin, Alisa Koch, Phillip Campbell, Geoffroy Laumet, Christian S. Stohler, Robert Dantzer, Jon-Kar Zubieta

**Affiliations:** 1 Department of Psychiatry and Behavioral Sciences, University of Texas McGovern Medical School, Houston, TX USA; 2 Translational Imaging Center, Houston Methodist Research Institute, Weill Cornell College of Medicine, Houston, TX USA; 3 Department of Internal Medicine, Division of Rheumatology, University of Michigan Medical School, Ann Arbor, MI USA; 4 Department of Physiology, Michigan State University, East Lansing, MI USA; 5 College of Dental Medicine, Columbia University, New York, NY USA; 6 Department of Symptom Research, Division of Internal Medicine, University of Texas MD Anderson Cancer Center, Houston, TX USA; 7 Department of Psychiatry, John T Mather Memorial Hospital, Northwell Health, Port Jefferson, NY USA

**Keywords:** Physiology, Psychology, Predictive markers, Neuroscience

## Abstract

Behavioral conditioning and expectation can have profound impact on animal and human physiology. Placebo, administered under positive expectation in clinical trials, can have potent effects on disease pathology, obscuring active medications. Emerging evidence suggests placebo-responsive neurotransmitter systems (e.g., endogenous opioid) regulate immune function by manipulating inflammatory proteins including IL-18, a potent pro-inflammatory, nociceptive cytokine implicated in pathophysiology of various diseases. Validation that neuroimmune interactions involving brain μ-opioid receptor (MOR) activity and plasma IL-18 underlie placebo analgesic expectation could have widespread clinical applications. Unfortunately, current lack of mechanistic clarity obfuscates clinical translation. To elucidate neuroimmune interactions underlying placebo analgesia, we exposed 37 healthy human volunteers to a standardized pain challenge on each of 2 days within a Positron Emission Tomography (PET) neuroimaging paradigm using the MOR selective radiotracer, ^11^C-Carfentanil (CFN). Each day volunteers received an intervention (placebo under analgesic expectation or no treatment), completed PET scanning, and rated their pain experience. MOR BP_ND_ parametric maps were generated from PET scans using standard methods. Results showed placebo reduced plasma IL-18 during pain (W_74_ = −3.7, *p* < 0.001), the extent correlating with reduction in pain scores. Placebo reduction in IL-18 covaried with placebo-induced endogenous opioid release in the left nucleus accumbens (T_148_ = 3.33; p_uncorr_ < 0.001) and left amygdala (T_148_ = 3.30; p_uncorr_ < 0.001). These findings are consistent with a modulating effect of placebo (under analgesic expectation in humans) on a potent nociceptive, pro-inflammatory cytokine (IL-18) and underlying relationships with endogenous opioid activity, a neurotransmitter system critically involved in pain, stress, and mood regulation.

## Introduction

Behavioral expectation and conditioning can have profound effects across various medical illnesses including affective disorders [1], irritable bowel syndrome [2], asthma [3], osteoarthritic pain [4], and acute and chronic pain states [5–12]. Administered under positive expectation, placebo can parallel or obscure effects of active medications in clinical trials [13, 14]. However, placebo effects have substantial inter-individual variability potentially linked to their impact on neurotransmitter function (e.g., dopamine, endogenous opioid, endocannabinoid, cholecystokinin) including genetic polymorphisms capable of modulating these neurotransmitter systems [2, 7, 15–17]. Current theories imply that placebo can interact with active medications synergistically, in a disease-modifying fashion [13, 18].

Research highlighting potential disease-modifying effects suggests placebo-responsive neurotransmitter systems can regulate immune function. Peripheral concentrations of interleukin (IL)−1β were selectively linked with endogenous opioid responses in the amygdala in both animal models [19] and humans [20]. Another study showed experimental mood induction regulated plasma IL-18, a pro-inflammatory IL-1 family cytokine implicated in the pathophysiology of various medical illnesses [21–28]. Induction of sad mood state increased (and neutral mood reduced) IL-18. Mood-induced regulation of IL-18 covaried with µ-opioid receptor (µOR) availability and endogenous opioid release in the nucleus accumbens and amygdala [29–31] during neutral (and sustained sad) affective states. Furthermore, these data suggest bi-directional relationships exist between central neurotransmitters modifiable by placebo administration (e.g., endogenous opioid) and stress-reactive innate immune processes. Table 1.

**Table 1:** Pain Measures.

Measure	No Placebo (mean +/− 1 SD)	Placebo (mean +/− 1 SD)	Wilcoxon W testing (Wilcoxon W, *p* value)
Pain Sensitivity	1.29 +/− 1	0.80 +/− 0.76	W74 = −2.1, *p* = 0.03
MPQ Sensory Pain	16 +/− 7	14 +/− 8	W74 = −1.5, *p* > 0.10
MPQ Affective Pain	2 +/− 2	1 +/− 2	W74 = −1.8, *p* = 0.07

Shown in the table are pain measures recorded from the pain challenge and obtained separately for each pre-treatment intervention (e.g., no intervention, placebo intervention). These measures include Pain sensitivity, the average pain intensity measure acquired during the 20 min sustained pain challenge over the volume of hypertonic saline (ml) needed to maintain a constant level of pain over time 47 and the McGill Pain Questionnaire scores which include Sensory and Affective pain scores. All measures were completed following the pain challenge on each of 2 days, the day without placebo pre-treatment and the day with placebo pre-treatment. Here the mean values (+/−1 SD) for each measure are reported in column 2 (no placebo) and column 3 (placebo) of the table. Repeated measures testing (using Wilcoxon W) compared means for each measure on the day of placebo pre-treatment with the respective means on the day with no placebo pre-treatment. Results (Wilcoxon W and un-corrected *p* value of significance) are reported in column 4. Significance was set to *p* < 0.05 to control for a type 1 error. Results indicated that placebo significantly reduced Pain Sensitivity and MPQ Affective Pain (marginal significance), but not MPQ Sensory Pain scores.

Studies in both animal models and humans manipulated expectation/conditioning paradigms to investigate placebo’s impact on specific immune factors. Pairing a gustatory stimulus (conditioned stimulus/CS) with cyclosporine A (immunosuppressive drug: unconditioned stimulus/US) showed that re-exposure to the CS alone mimicked immunopharmacological properties of cyclosporine A, evidenced by impaired Th1 cytokine production and decreased T cell proliferation [32, 33]. Repeated pairing of the CS and US, but not single pairings, or simple manipulation of expectations, induced “placebo-elicited” changes in specific inflammatory proteins (e.g., IL-2, IL-6, IL-10, TNF-a) [34–38].

While exact mechanisms remain unclear, evidence suggests central opioid neurotransmitters interact with and potentially regulate peripheral inflammatory processes involving IL-1 family cytokines (e.g., IL-1β, IL-18) [20, 29–31]. That IL-18 is further implicated disease pathophysiology [21–28] underscores the clinical translational relevance of these neuroimmune interactions. Here, we examined whether administration of placebo (intravenous isotonic saline in volunteer sight) under expectation of analgesia modulates plasma IL-18. We hypothesized that placebo’s immune modulation would be proportional to endogenous opioid activity in brain regions that integrate input from viscera and peripheral nociceptive fibers [39] and immune processes (i.e., central amygdala) [40], modulate outflow to the peripheral immune system (e.g., hypothalamus, amygdala) [30, 41, 42], encode reward expectation and salience (e.g., nucleus accumbens) [9, 43], and where we previously identified neuroimmune interactions involving IL-1 family cytokines and µOR activation during negative affective states [20, 29–31].

## Methods

**Fig. 1:**
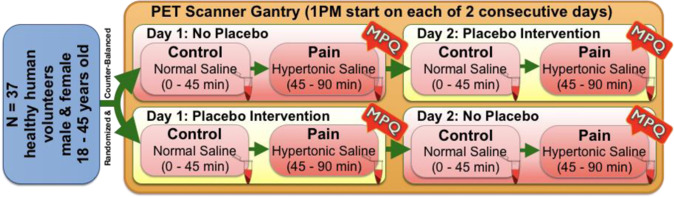
Research paradigm. The sample derived from *n* = 37 healthy volunteers (12 males, 25 females). Once enrolled, volunteers were randomized and counterbalanced to 1 of 2 intervention orders as illustrated by the green double arrows on the left. As such, a given subject was randomized to either Order #1: No Placebo on Day 1 and Placebo Intervention on day 2 (see top row of diagram) or Order #2: Placebo intervention on Day 1 and No Placebo on Day 2 (bottom row of diagram). Regarding Placebo/NoPlacebo administration, subjects were told that on one of the two scanning days they will receive an intravenous dose of “an agent that may increase the body’s ability to counter pain” and that on the other scanning day they will receive no intervention (e.g., an inert substance). After completion of the studies, the volunteers were informed that the agent was a placebo, an inactive agent.

This research was approved by Institutional Review Boards and Radioactive Drug Research Committees at the University of Michigan and the University of Texas McGovern Medical School. Written informed consent was obtained from all volunteers. The overall research paradigm is depicted in Fig. 1.

### Subjects

A total of 37 right-handed, healthy, non-smoker, male (*n* = 12) and female (*n* = 25) volunteers completed positron emission tomography (PET) brain scans with [^11^C]-carfentanil (CFN), a radiotracer with specific binding at the µOR [44]. Study volunteers had negative urine drug screens and no medical or psychiatric illness (Structured Clinical Interview for DSM-IV) [45]. Female volunteers reported regular menstrual cycles (28–32 days), negative urine pregnancy testing, and completed PET scanning between day 2 and 10 post menses. No volunteers were using psychotropic/hormonal medications or compounds with known effects on IL-1 family cytokines or opioid mechanisms for 6 months prior. In prior studies, NEO-PI Neuroticism was associated with immune measures [20], so volunteers completed a personality inventory (NEO PI-R) [46]. Additional measures obtained at study entry (Age, Body Mass Index [BMI: kg/m^2^]) were tested for effects on IL-18.

### General study procedure

Two serial 90 min [^11^C]-CFN PET scans were conducted on separate days, with or without placebo treatment as previously described [7, 8]. The current dataset (acquired between 2007 and 2012) is distinct from the previously described dataset [7, 8]. Treatment order was randomized and counterbalanced. Placebo treatment involved intravenous injection of 1 mL of 0.9% saline in volunteer sight every 4 min during PET scanning, described as “an agent that may increase the body’s ability to counter pain”. On the day without Placebo, subjects were told they would not receive an analgesic treatment. PET scans began at 1:00 PM (+/− 30 min) and included two 45-minute segments, control (0–45 min) and pain (45–90 min) conditions as illustrated in Fig. 1. Prior to each PET scan, two intravenous catheters were placed in antecubital veins for radiotracer administration and blood sampling, permitting venous blood collection in heparinized tubes every 10 min. Upon completion, subjects were informed the “potential pain medication” was placebo.

### Experimental pain challenge

The pain challenge involved a non-painful “control” condition (pain is rated but not delivered) and a sustained pain condition [8, 47]. The control condition (applied at 0 min) involved intramuscular (masseter) injection of non-painful 0.9% saline over 20 min. The pain condition (applied at 45 min) involved intramuscular (masseter) injection of medication-grade 5% hypertonic saline over 20 min via a computer-controlled delivery system. The computer recorded subject pain intensity every 15 s using a visual analog scale (VAS) (0: no pain; 100: most pain imaginable) subsequently altering hypertonic saline delivery to target a 40% maximum pain intensity as previously described [48, 49]. Order of conditions (control, pain) was maintained to avoid carry-over effects.

Volume of hypertonic saline required for pain maintenance was recorded, providing an objective measure of Pain Sensitivity for each condition (no treatment, placebo treatment) [48, 49]. Following completion of the challenges, pain was quantified using the McGill Pain Questionnaire (MPQ) [50] yielding MPQ Sensory and Affective Pain scores.

Expectations of placebo analgesia were quantified with a 0–100 VAS scale (0 = no expectation, 100 = expecting complete relief). Subjective assessments of placebo analgesia (0 = no relief, 100 = complete relief) were after placebo scans.

### Neuroimaging measures

Central μOR availability (non-displaceable binding potential, BP_ND_) was quantified in vivo as described [47]. Scans were acquired using a Siemens HR^+^ scanner (Knoxville, TN) in 3-D mode (FWHM resolution 5.5 mm in-plane and 5.0 mm axially). Synthesis of ^11^C-CFN, image acquisition, co-registration, and reconstruction were as previously described [7, 51]. Maximum mass of CFN was 0.03 µg/kg, ensuring sub-pharmacological tracer doses. Injected doses were 15 ± 1 mCi (555 ± 37 MBq), 50% administered over 10 s and 50% infused over the entire scan to rapidly achieve/maintain steady state. Dynamic images were transformed into two sets of co-registered parametric maps: (a) a tracer transport measure (*K*_1_ ratio) and (b) BP_ND_, generated using modified Logan analyses [52] with occipital cortex (devoid of µOR) as reference region. Approximately 5–7 min after tracer administration, the Logan plot becomes linear with slope proportional to BP_ND_ (BP_ND_ = [B_max_/K_d_] + 1; *B*_*max*_ = receptor concentration; *K*_*d*_ = receptor affinity). μOR BP_ND_ was calculated for control (0–45 min), control + placebo (0–45 min), pain (45–90 min), and pain + placebo (45–90 min) conditions. Structural T1 images, acquired via 3 Tesla MRI scanner (Signa, General Electric, WI) enabled anatomical co-registration and normalization to standardized stereotactic coordinates [47].

### Measures of inflammation

Whole blood was obtained from indwelling IV catheter, immediately placed on ice, centrifuged within 30 min for 15 min at 4000 rpm, separated, and plasma aliquots stored at −80 °C. Sample storage time averaged ~3–4 years. Plasma IL-18 concentration was quantified from samples at 45 min (control condition) and 90 min (pain condition) for each of treatment condition (placebo, no placebo) via standard ELISA using IL-18 kits (R&D Systems, MN), samples run in duplicate. To determine assay sensitivity, we assayed serially diluted human IL-18 calibrator. Mean absorbance + 2SD for calibrator diluted to 6.25 pg/ml was lower than mean absorbance −2SD for calibrator diluted to 12.5 pg/ml (minimal detectable concentration). Average intra-assay coefficient of variation was calculated to be 3.9%. The highest standard was 1000 pg/ml. IL-18 concentration from each sample was averaged across duplicate pairs.

### Planned analyses

Psychophysical data (Age, BMI, NEO-PI Neuroticism, pain measures) and IL-18 were substantially skewed and/or not continuous, violating normality assumptions. These data were analyzed using non-parametric techniques. Mann–Whitney *U* testing confirmed distributions of psychophysical variables did not differ across sexes. Using IL-18 during the non-painful, control condition we tested both for sex differences in IL-18 (Mann–Whitney *U*) and correlations (Spearman) between IL-18 and potential confounders (age, Neuroticism, BMI). Separate Wilcoxon signed rank paired testing showed the effect of pain on plasma IL-18 and the effect of placebo on plasma IL-18. Spearman Rank correlation tested for relationships between pain related variables (changes in IL-18, Pain Sensitivity, MPQ Sensory and Affective scores). Significance for each was set to *p* < 0.05 to control for a type 1 error.

### Imaging analyses

involved whole brain voxel-by-voxel ANCOVA testing using SPM12 (Wellcome Trust, England) and MATLAB (Mathworks, MA). Imaging data included control μOR BP_ND_ and placebo induced activation of µOR (quantified as change in μOR BP_ND_ during control and pain with placebo administration). ANCOVA tested for linear relationships between IL-18 (covariate) and µOR BP_ND_ (outcome) during the control condition. Repeated Measures ANCOVA tested for linear relationships between these variables across conditions (i.e., placebo-induced changes during control, pain). Causality isn’t inferred from results presented; ANCOVA results are presented as associational only. Based on a-priori hypotheses, we anticipated significant linear relationships between two measures (IL-18, µOR BP_ND_) would occur in a regionally specific manner (amygdala, nucleus accumbens) [20, 29–31, 53], after correction for multiple comparisons. Significance was set at *p* < 0.001 for a priori hypothesized regions and FDR-corrected *p* < 0.05 with a 10-voxel minimum for other regions to control for a type I error.

## Results

### Demographic and psychophysical characteristics

The study included 37 healthy humans. Mann–Whitney *U* testing showed no differences in baseline psychophysical variables (e.g., Age, BMI, NEO-PI Neuroticism) between males and females (*P* > 0.10 for each).

### Relationships between µOR BP_ND_ and IL-18 concentrations during control condition

**Fig. 2:**
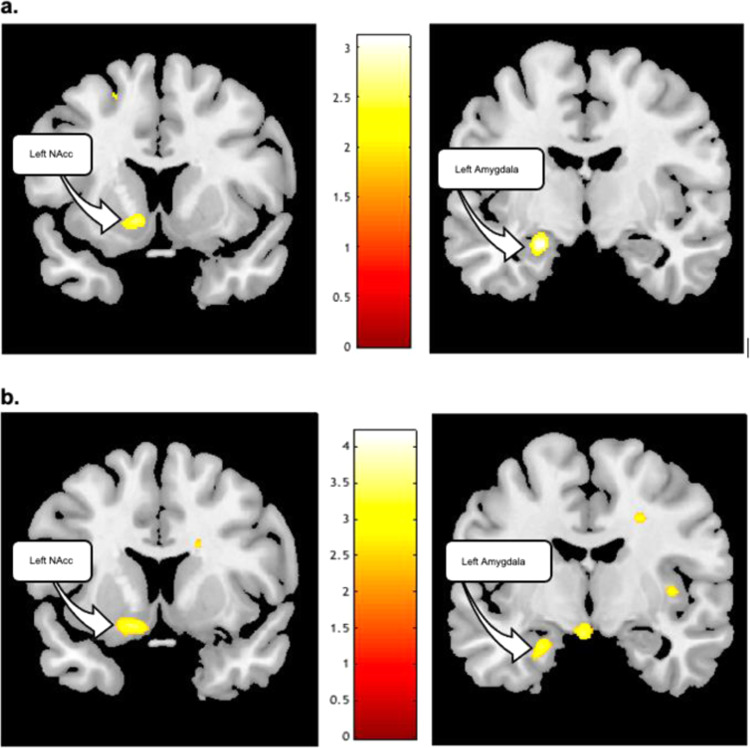
Relationships between endogenous opioid neurotransmitter activity and plasma IL-18 concentrations during the control condition. In this figure we illustrate these relationships both without placebo intervention (Panel (**a**) top of Fig. 2) and in response to placebo intervention (Panel (**b**) bottom of Fig. 2). **a** Relationship between µ-opioid receptor BPND and plasma concentration of IL-18 during the control condition (e.g., pain anticipation) without placebo administration. The sample derived from *n* = 37 healthy volunteers (12 males, 25 females). Significance was set to *p* < 0.001 and at least 10 contiguous voxels for regions with a priori regional hypotheses. Correlations reached the threshold for significance in left nucleus accumbens (xyz = −11, 11, −6; T35 = −2.7; puncorr < 0.005) and left amygdala (xyz = −23, −8, −17; T35 = −3.1; puncorr < 0.001). Color blobs within brain images represent regional *T* scores whose values are described in the color bar (inset), ranging from *T* = 0 (dark red) to *T* > 3 (yellow/white). **b** Relationship between changes in µ-opioid receptor BPND before and after placebo administration and changes in plasma concentrations of IL-18 during the pain challenge. The sample derived from *n* = 37 healthy volunteers (12 males, 25 females). Significance was set to *p* < 0.001 and at least 10 contiguous voxels for regions with a priori regional hypotheses. Significant effects were observed in the left nucleus accumbens (xyz = −12, 10, −13; T148 = 3.33; puncorr < 0.001), and left amygdala (xyz = −23, −7, −22; T148 = 3.30; puncorr < 0.001. Color blobs within brain images represent regional *T* scores whose values are described in the color bar (inset), ranging from *T* = 0 (dark red) to *T* > 3 (yellow/white).

No significant relationships were observed between IL-18 and age (rho = 0.21, *p* > 0.10) or BMI (rho = 0.20, *p* > 0.10). There were no sex differences in plasma IL-18 (U_37_ = 0.6; *p* > 0.1; males (235 +/− 171 pg/ml), females (198 +/− 137 pg/ml). Whole brain, voxel-by-voxel ANCOVA analyses showed plasma IL-18 covaried inversely with µOR BP_ND_ during the control condition. Significant effects localized to the left nucleus accumbens (xyz = −11, 11, −6; T_35_ = −2.7; p_uncorr_ < 0.005; marginally significant) and left amygdala (xyz = −23, −8, −17; T_35_ = −3.1; p_uncorr_ < 0.001) (Fig. 2a).

### Effects of sustained pain on plasma IL-18

No sex differences in MPQ Sensory or Affective scores or Pain Sensitivity were observed (Mann–Whiney *U*, *p* > 0.10 for each). Plasma IL-18 was significantly reduced during the pain challenge (Wilcoxon signed rank paired testing W_74_ = −2.0, *p* < 0.05) by an average of 12 pg/ml (6%) compared to control condition. Pain-induced reduction in IL-18 correlated with reduction in MPQ affective pain scores (rho = 0.31, *p* = 0.03), but not with MPQ sensory pain scores (rho = 0.10, *p* > 0.10) or Pain Sensitivity (rho = 0.18, *p* > 0.10).

### Effect of placebo during control condition

**Fig. 3:**
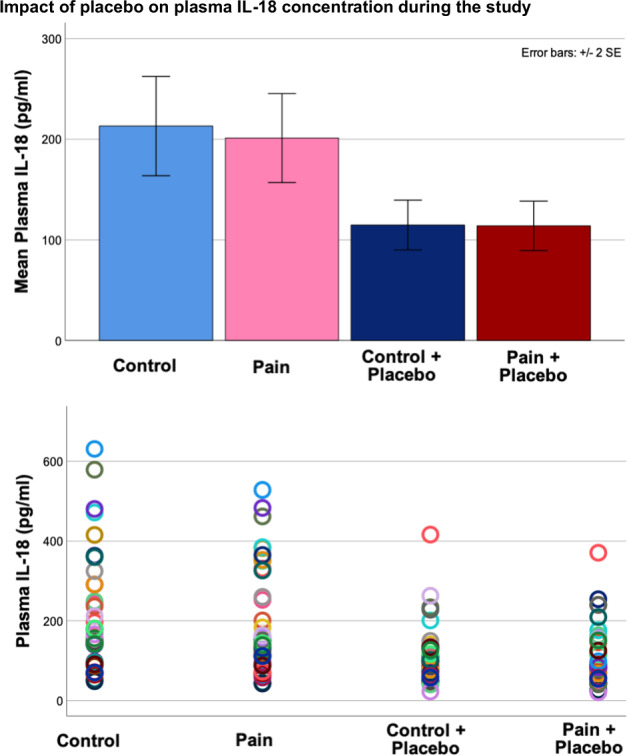
Impact of placebo on plasma Il-18 concentration during the study. The sample comprises *n* = 37 healthy volunteers (12 males, 25 females). Illustrated in the color bar graphs (top) are mean plasma IL-18 concentrations (+/− 2 SE). Colored in blue are mean IL-18 concentrations during the control condition without placebo intervention (light blue color) and with placebo intervention (dark blue). Colored in pink/red are mean IL-18 concentrations following sustained pain without placebo intervention (light pink) and with placebo intervention (dark red). Illustrated in the colored dot plots (bottom) are subjects’ IL-18 concentrations (vertical axis) separated by experimental condition (Control, Pain, Control + Placebo, Pain + Placebo) on the horizontal axis. Each subject is represented by a specific color. Placebo treatment significantly reduced plasma IL-18 concentration during pain anticipation (W74 = − 3.5, *p* < 0.001), reflecting a mean IL-18 reduction of 97 pg/ml (46%) following placebo administration.

Placebo significantly reduced plasma IL-18 (Wilcoxon signed rank paired testing, W_74_ = −3.5, *p* < 0.001: mean IL-18 reduction 97 pg/ml, 46%) (Fig. 3).

We tested for relationships between placebo-induced changes in plasma IL-18 and endogenous opioid activation (change in µOR BP_ND_ from control to control + placebo) using repeated measures ANCOVA. No significant effects were observed between placebo-induced reductions in plasma IL-18 and placebo-induced activation of endogenous opioid neurotransmission during the control conditions (*p* > 0.001).

### Effect of placebo during sustained pain challenge

Wilcoxon signed rank paired testing showed placebo had significant effects, reducing Pain Sensitivity by 17% (W_74_ = −2.1, *p* = 0.03), marginally reducing MPQ Affective scores, by 18% (W_74_ = −1.8, *p* = 0.07), but not MPQ Sensory scores (W_74_ = −1.5, *p* = 0.14).

Expectations of placebo analgesia correlated with placebo reduction in MPQ Affective pain (rho = −0.34, *p* = 0.02) and MPQ Sensory pain scores (rho = −0.30, *p* = 0.03), but not with Pain Sensitivity (rho = −0.10, *p* > 0.10). Self-reported analgesic effectiveness correlated with placebo reduction in pain sensitivity (rho = −0.69, *p* < 0.001), but not with either MPQ Affective (rho = −0.16, *p* > 0.10) or MPQ Sensory pain scores (rho = −0.07, *p* > 0.10).

**Fig. 4:**
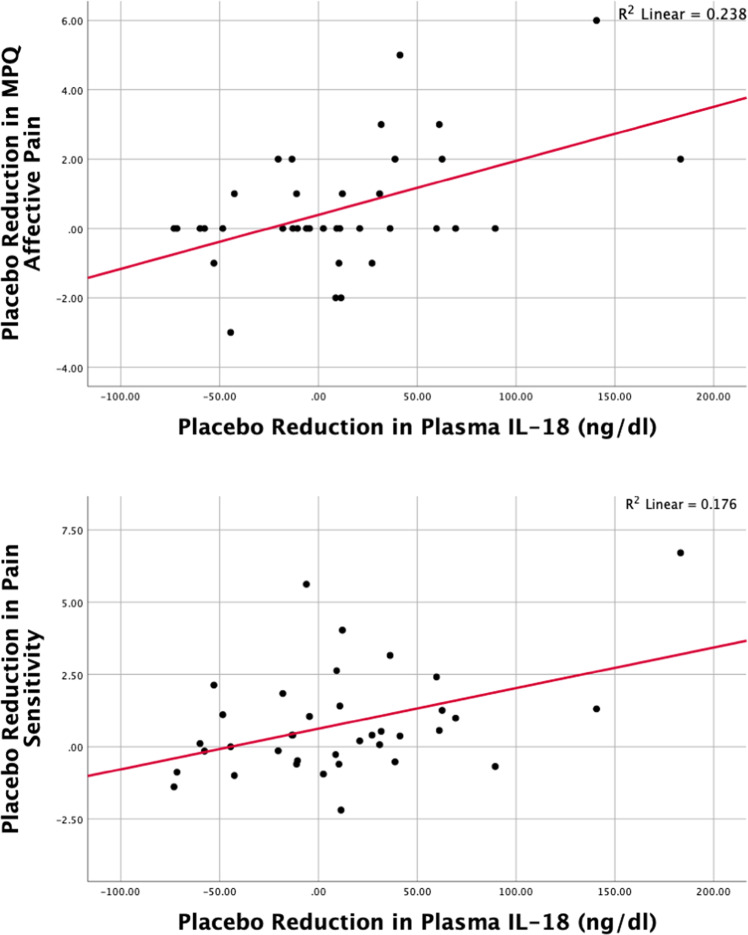
Relationships between placebo-induced changes in MPQ affective pain (top graph) and pain sensitivity scores (bottom graph) with placebo induced changes in plasma IL-18 are depicted in this graph. The sample derived from *n* = 37 healthy volunteers (12 males, 25 females). (Note: correlation testing with placebo-induced changes in MPQ Sensory Pain score was not completed given the lack of significance in testing of correlations between MPQ Sensory Pain and pain induced change in plasma IL-18). The top graph shows the correlation between placebo-induced reductions in MPQ Affective pain scores and placebo-induced changes in IL-18 plasma levels during the pain challenge (*r* = −0.49, *p* = 0.001). The bottom graph shows similar relationships for placebo induced changes in Pain Sensitivity and IL-18 during pain (*r* = −0.42, *p* < 0.005).

Significant reductions in IL-18 were observed from pain to pain + placebo conditions (W_74_ = −3.7, *p* < 0.001), the extent correlating with placebo-induced reductions in Pain Sensitivity (rho = 0.33, *p* < 0.05), MPQ Affective pain scores (rho = 0.43, *p* = 0.008), and MPQ Sensory pain scores (rho = 0.33, *p* < 0.05) (Fig. 4). Whole brain, voxel-by-voxel repeated measures ANCOVA between plasma IL-18 and endogenous opioid activity showed placebo-induced reductions in IL-18 covaried with placebo-induced endogenous opioid release in the left nucleus accumbens (xyz = −12, 10, −13; T_148_ = 3.33; p_uncorr_ < 0.001) and left amygdala (xyz = −23, −7, −22; T_148_ = 3.30; p_uncorr_ < 0.001) (Fig. 2b).

## Discussion

We previously showed, in healthy humans during a non-painful, control condition, that plasma IL-1β was inversely proportional to µOR BP_ND_ in the amygdala bilaterally. Subsequently, sustained pain induced increase in anti-nociceptive IL-1ra covaried with endogenous opioid release in the nucleus accumbens, an effect moderated by pro-nociceptive IL-1β, suggesting strong interactions between central endogenous opioid neurotransmitter mechanisms and inflammatory mechanisms [20]. Here we present new data identifying significant neuroimmune interactions between endogenous opioid neurotransmitter activity and another IL-1 family cytokine, IL-18.

IL-18 is a potent pro-nociceptive [54–58], pro-inflammatory, IL-1 family cytokine [59] with morphine tolerance inducing effects [57, 60]. Plasma IL-18 is regulated in part by binding of endogenous opioid neurotransmitters to surface toll-like receptor-4 on immune cells [61, 62]. Inactive IL-18 is constitutively expressed in cells throughout the body, many being highly reactive to psychosocial stressors. Inactive “pro-IL-18” remains in the cytosol pending activation, a process involving cleavage of pro-IL-18 via caspase-1 enzyme. Once activated, IL-18 can readily exit its constituent cell. In anterior pituitary cells, active IL-18 [63, 64] readily exits the cell to enter the circulating blood, providing an explanation for how brain opioids can alter plasma concentration of IL-18. Plasma IL-18 can be modulated by exposure to brief behavioral challenges in both animal models [65, 66] and humans [29–31]. In addition, peripheral IL-1 family cytokines can impact brain neurotransmission by an indirect route via activation of vagal afferents which, via neuronal projections to the nucleus of the tractus solitarius and amygdala, can impact stress-reactive neurotransmission [67].

In healthy volunteers resting during the control condition (no pain, no placebo pre-treatment), we showed that plasma IL-18 concentration varied inversely with brain µOR availability (possibly reflecting basal opioid tone [68]) in the left amygdala and left nucleus accumbens. Current understanding of central opioid – plasma cytokine neuroimmune interactions suggests it’s possible that neuroimmune homeostasis is maintained during relatively non-stressful events (e.g., control condition) in part via bi-directional interactions between neuronal processes (e.g., endogenous opioid tone in the Amygdala) and peripheral inflammatory protein activation (plasma IL-18), which vary linearly in our sample during the restful control condition. Future studies quantifying vagal afferent activity may better elucidate these bi-directional relationships.

During the pain challenge we showed a small but significant reduction in plasma IL-18 which covaried with MPQ affective pain but not MPQ sensory pain scores. This was unexpected as we anticipated pain-induced plasma IL-18 elevation. However, in retrospect, given that study subjects are otherwise healthy, without acute or chronic pain, and for whom resolution of pain occurred shortly after the challenge, it is possible that physiological mechanisms in these healthy subjects were not overwhelmed during the experimental challenge. Furthermore, these potential physiological mechanisms appear adequately functioning, reducing a potent nociceptive protein (IL-18) and potentially reducing persistence of pain in these healthy individuals. Evidence from animal models suggests that under physiological conditions and in response to certain stressors, effects of IL-18 are buffered by another potentially stress-reactive, endogenous protein, IL-18 binding protein (IL-18bp) [69]. While we have not quantified IL-18bp in the current study, we identified a reduction in IL-18, suggesting there was a concurrent increase in IL-18bp, buffering the potent pain inducing effects of IL-18.

We showed that administration of placebo (with expectation of analgesia) significantly reduced plasma concentration of IL-18 by 46% during the control condition. Indeed, IL-18 is implicated in emergence of hyperalgesia [70, 71] and morphine tolerance [60]. We also showed extent of subjects’ expectation of placebo analgesia (quantified prior to pain challenge) significantly correlated with MPQ Affective and Sensory pain scores. Taken together, these findings suggest expectation of placebo analgesia, via reduction in pro-nociceptive IL-1 family cytokines (e.g., IL-18) during the control condition, may set the stage for reduction in MPQ pain scores quantified during the pain challenge.

Placebo significantly reduced plasma IL-18 during the pain condition. The magnitude of IL-18 reduction correlated with the magnitude of reduction in MPQ Affective and Sensory pain scores and an objective measure, Pain Sensitivity. Furthermore, placebo-induced reduction in IL-18 covaried with placebo induced endogenous opioid release in the left nucleus accumbens and left amygdala. As noted in a review by Vits and colleagues [36], A prerequisite for the classical conditioning of immune functions is the functional interaction between the central nervous system (CNS) and the peripheral immune system [72–74]. In their review, Vits and colleagues list the autonomic nervous system as a key pathway for communication between the Central Nervous System (CNS) and the peripheral immune system during conditioned immunosuppression. However, despite this and other encouraging research [75], the role of CNS neurotransmitters (and brain regional localization) in behavioral conditioning of immune function (and in immune modulation during human placebo expectation) remain incompletely understood. To our knowledge, this is the first in-vivo human study to identify mind-body interactions potentially underlying potent analgesic effects of placebo. Here, during the control condition, whole brain, voxel by voxel ANCOVA showed placebo-induced change in plasma IL-18 did not covary with placebo-induced change in µOR availability. In contrast, during the pain challenge, prominent covariant effects were identified between placebo-induced reduction in plasma IL-18 and placebo-induced endogenous opioid release, interactions during a more stressful, painful state.

Note that placebo-induced neuroimmune interactions identified during the pain condition localized to the Amygdala and Nucleus Accumbens, the same brain regions wherein we previously identified interactions between plasma IL-18 and brain endogenous opioid release during induction of a negative affective state. This finding is consistent with our original hypothesis that magnitude of placebo’s immune modulating effects are proportional to endogenous opioid release within brain regions that integrate input from viscera and peripheral nociceptive fibers [39] and immune processes [40] (central amygdala), modulate outflow to the peripheral immune system (hypothalamus, amygdala) [48, 59–62] and encode reward expectation and salience (nucleus accumbens) [9, 43]. However, mechanisms underlying lateralization of imaging findings (and clinical impact on pain) are unclear. Strict lateralization of brain effects was not present in our prior studies of placebo-induced opioidergic activity [7, 8] or neuroimmune interactions during experimental mood induction [29–31]. Future studies specifically probing factors underlying lateralization effects would help clarify these issues.

While the results presented are novel and provide evidence supporting development of innovative, personalized expectation-based interventions, certain factors limit the extent of translation to treatment of clinical pain states. Subjects included in the current study were healthy, without acute/chronic medical illness including pain, so our finding may reflect normal physiologic processes. That these findings were present in the absence of disease further underscores their importance in informing understanding of currently unknown, mechanisms potentially underlying placebo effect in general and placebo analgesia in particular. However, the underlying study design isn’t optimal to inform on functional relevance. Expansion of investigation in patient populations (within a paradigm developed to inform functional relevance) would help elucidate functional translation of our findings to disease pathology. Additionally, given data skewing, we used non-parametric methods. We recognize that, in presence of normally distributed data and adequate sample size, implementing ANOVA/ANCOVA analyses with a 2x2 factorial design could permit more robust interaction analyses involving covariates. Finally, while our experimental strategy created a condition of placebo expectation, we didn’t control for effect (without expectation) of so-called vehicle. In contrast to the placebo condition where normal saline was injected in full view of the subjects (described as an injection of analgesic) to elicit expectation of placebo analgesia, during the control condition, no injection was given (in full view of subjects) to limit subjects’ expectation of analgesia. Control for the vehicle via a third condition wherein normal saline was injected and described as an inert substance with no related analgesia may further isolate effects related to expectation. Other strategies employing patient blinding are worthy of consideration for future studies. In addition, variation in sample storage time could impact inter-individual comparisons. However, all samples for each subject were stored for the same duration. Given our primary focus on testing within subject effects, we don’t expect inter-subject variation in sample storage confounded these findings.
